# Occurrence of multipolar mitoses and association with Aurora-A/-B kinases and p53 mutations in aneuploid esophageal carcinoma cells

**DOI:** 10.1186/1471-2121-12-13

**Published:** 2011-04-06

**Authors:** Christiane D Fichter, Corinna Herz, Claudia Münch, Oliver G Opitz, Martin Werner, Silke Lassmann

**Affiliations:** 1 Institute of Pathology, University Medical Center, Freiburg, Germany; 2 Tumorzentrum Ludwig Heilmeyer - Comprehensive Cancer Center, University Medical Center, Freiburg, Germany; 3 Dept. of Environmental Health Sciences, University Medical Center Freiburg, Germany

## Abstract

**Background:**

Aurora kinases and loss of p53 function are implicated in the carcinogenesis of aneuploid esophageal cancers. Their association with occurrence of multipolar mitoses in the two main histotypes of aneuploid esophageal squamous cell carcinoma (ESCC) and Barrett's adenocarcinoma (BAC) remains unclear. Here, we investigated the occurrence of multipolar mitoses, Aurora-A/-B gene copy numbers and expression/activation as well as p53 alterations in aneuploid ESCC and BAC cancer cell lines.

**Results:**

A control esophageal epithelial cell line (EPC-hTERT) had normal Aurora-A and -B gene copy numbers and expression, was p53 wild type and displayed bipolar mitoses. In contrast, both ESCC (OE21, Kyse-410) and BAC (OE33, OE19) cell lines were aneuploid and displayed elevated gene copy numbers of Aurora-A (chromosome 20 polysomy: OE21, OE33, OE19; gene amplification: Kyse-410) and Aurora-B (chromosome 17 polysomy: OE21, Kyse-410). Aurora-B gene copy numbers were not elevated in OE19 and OE33 cells despite chromosome 17 polysomy. Aurora-A expression and activity (Aurora-A/phosphoT288) was not directly linked to gene copy numbers and was highest in Kyse-410 and OE33 cells. Aurora-B expression and activity (Aurora-B/phosphoT232) was higher in OE21 and Kyse-410 than in OE33 and OE19 cells. The mitotic index was highest in OE21, followed by OE33 > OE19 > Kyse-410 and EPC-hTERT cells. Multipolar mitoses occurred with high frequency in OE33 (13.8 ± 4.2%), followed by OE21 (7.7 ± 5.0%) and Kyse-410 (6.3 ± 2.0%) cells. Single multipolar mitoses occurred in OE19 (1.0 ± 1.0%) cells. Distinct p53 mutations and p53 protein expression patterns were found in all esophageal cancer cell lines, but complete functional p53 inactivation occurred in OE21 and OE33 only.

**Conclusions:**

High Aurora-A expression alone is not associated with overt multipolar mitoses in aneuploid ESCC and BAC cancer cells, as specifically shown here for OE21 and OE33 cells, respectively. Additional p53 loss of function mutations are necessary for this to occur, at least for invasive esophageal cancer cells. Further assessment of Aurora kinases and p53 interactions in cells or tissue specimens derived from non-invasive dysplasia (ESCC) or intestinal metaplasia (BAC) are necessary to disclose a potential causative role of Aurora kinases and p53 for development of aneuploid, invasive esophageal cancers.

## Background

Esophageal cancer is one of the leading causes of death from cancers worldwide. The two major histotypes of esophageal cancer are esophageal squamous cell carcinoma (ESCC) and Barrett's adenocarcinoma (BAC) [1,2].

Several specific molecular alterations play crucial roles in the carcinogenesis of ESCC or BAC, with tumor cell aneuploidy and p53 mutations being major hallmarks of both ESCC and BAC [3-5]. In fact, aneuploidy is found in 50% to 70% of ESCC and is associated with poor prognosis [6,7]. In BAC, similar high rates of aneuploidy are seen for invasive carcinomas [8,9], and aneuploidy is an early event in the metaplasia-dysplasia-adenocarcinoma sequence of BAC. Moreover, p53 is mutated in 35% to 80% of ESCC and in about 50% to 90% of BAC [4,10,11].

Together with deregulation of mitotic and post-mitotic cell cycle control points, the presence of supernumerary centrosomes has been proposed as one likely mechanism for development and/or maintenance of aneuploidy [12]. Supernumerary centrosomes have been detected in several aneuploid human cancers or cell lines derived thereof by evaluation of centrosomal proteins, such as γ-tubulin, pericentrin or Inhibitor of DNA binding protein 1 (ID1) [13-15]. However, the association of supernumerary centrosomes with multipolar mitoses in aneuploid ESCC and BAC cells has not been studied so far.

The Aurora kinase family of serine/threonine kinases regulates many processes during cell division and is currently discussed as therapeutic target in cancer [16,17]. Specifically, Aurora-A is important for centrosome maturation, separation and spindle assembly [16]. Amplification of the Aurora-A locus (*AURKA*, 20q13.2) and subsequent overexpression of Aurora-A was observed for example in colorectal [18] and pancreatic cancer [19], as well as in ESCCs and BACs [20-26]. Overexpression of Aurora-A has been functionally associated with supernumerary centrosomes and aneuploidy [27-31]. In esophageal cancers, a polymorphism of Aurora-A was associated with increased esophageal cancer risk. This Aurora-A polymorphism showed reduced Aurora-A kinase activity, lack of phosphorylation of its substrate Lats2 and associated genetic instability, at least by ectopic expression of the Aurora-A isoforms in immortalized fibroblasts [32]. Whether or not lack of Lats2 phosphorylation alone and/or other alterations of the Aurora-A isoforms, such as incorrect intracellular localization, are responsible for genomic instability in esophageal cancer cells remained elusive.

In contrast, Aurora-B is involved in kinetochore-microtubule interactions, chromosome condensation and cytokinesis [16]. Together with INCENP, survivin and borealin, Aurora-B builds the chromosomal passenger complex [33]. The Aurora-B gene (*AURKB*) is located in the chromosomal region 17p13.1 [16], which is also frequently altered in ESCCs and BACs [34-37]. Although the role of Aurora-B in human cancer is less clear than for Aurora-A, an association between Aurora-B overexpression and aneuploidy has been reported for some cancer cell lines [16,38]. However, in esophageal cancer the association of Aurora-A and Aurora-B with occurrence of multipolar mitoses in aneuploid ESCC or BAC cells remains elusive so far.

In view of the crucial role of the tumor suppressor p53 for maintenance of genetic stability [39,40] and its frequent mutation in esophageal cancer [4,10,11], it is of interest that also a centrosomal localization and functional involvement in centrosome duplication has been described for p53 [41-43]. Moreover, p53 can be phosphorylated by Aurora-A, leading to MDM2 dependent p53 inactivation and degradation and/or loss of p53 transactivation activity [44,45]. Together, (Aurora-A dependent or independent) disruption of p53 function may result in escape of the p53 dependent G1 post-mitotic checkpoint [46] and potentially also centrosomal dysfunction.

The aim of the present study was to investigate the occurrence of multipolar mitoses and association with Aurora kinases and p53 mutations in previously established esophageal carcinoma cell lines [47-49] and control esophageal epithelial cells [50-52].

## Results

### Ploidy and cell cycle distribution in normal esophageal epithelial cells and esophageal cancer cells

For the present study, a control diploid cell line derived from normal esophageal epithelial cells [50,51] as well as four aneuploid esophageal cancer cell lines with squamous cell (OE21, Kyse-410, hereafter referred to as ESCC) and adenocarcinoma (OE33, OE19, hereafter referred to as "BAC") differentiation and growth patterns [47-49,52], i.e. closely reflecting the morphological features of the two main histotypes of esophageal cancer, were used. All experimental data shown are derived from each three independent experiments.

**Figure 1:**
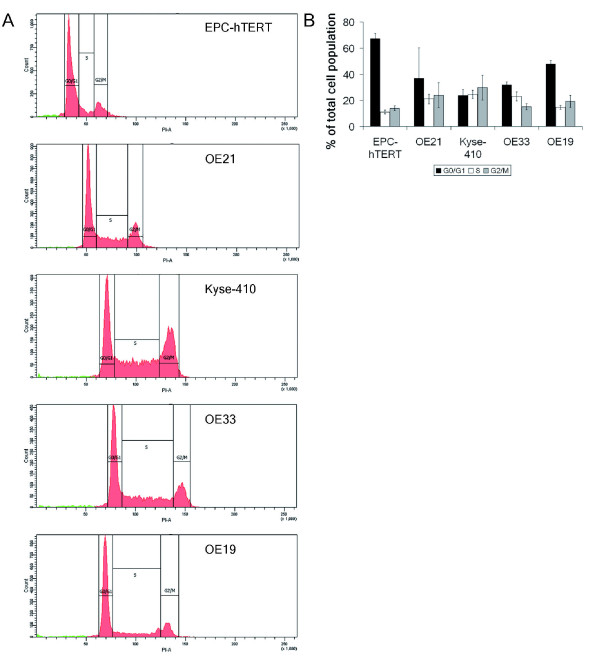
**Cell cycle phase distribution analysis by flow cytometry in normal esophageal epithelial cells and esophageal cancer cells**. **A**. The histograms show the cell cycle phase distribution, representative for 3 independent experiments. Note the increasing PI-staining/DNA content from EPC-hTERT cells to OE21, Kyse-410, OE33 and OE19 cells. Proliferating cells in red, apoptotic cells in green. **B**. The bar graph shows the distribution of cells in G0/G1-, S- and G2/M-phase of the cell cycle (mean and ± standard deviation of three independent experiments).

Ploidy, respective DNA content, as well as cell cycle distribution patterns of all five cell lines was first defined by flow cytometry. This validated diploidy of EPC-hTERT cells and aneuploidy to different levels in the esophageal cancer cell lines (Figure 1A). To further define chromosome numbers in the aneuploid esophageal cancer cell lines, each 10 metaphase spreads were analyzed and revealed highest chromosome numbers in OE33 (100.2 ± 4.4), followed by Kyse-410 (89.6 ± 5.1), OE21 (70.6 ± 2.5) and OE19 (56.8 ± 1.8) cells. Analyses of cell cycle distribution (Figure 1B) revealed that ESCC cells (OE21, Kyse-410) showed similarly distributed cell populations in G0/G1-, S- and G2/M-phases (each about 20-30%). In contrast, OE33 (~30%) and markedly OE19 (~50%) and EPC-hTERT (~70%) cells had a high G0/G1-phase population, with reduced S- and G2/M-phase populations.

### Aurora kinases in normal esophageal epithelial cells and esophageal cancer cells

**Figure 2:**
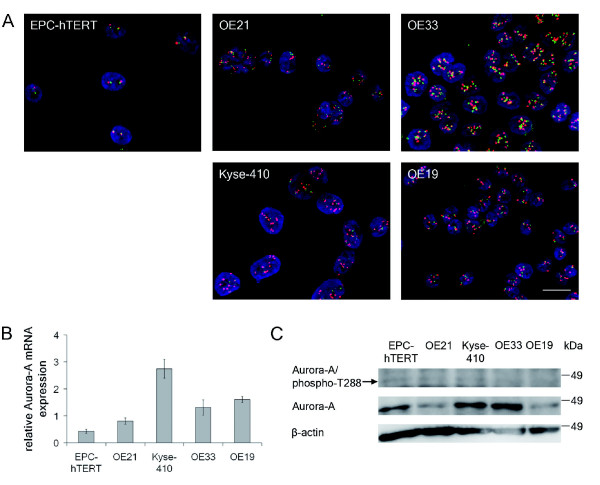
**Aurora-A gene copy numbers, mRNA and protein expression in normal esophageal epithelial cells and esophageal cancer cells**. **A**. FISH analysis of *AURKA *(red signals) and Chromosome 20 (CEP20; green signals). Bar = 20 μm, all panels are in the same magnification. **B**. Relative Aurora-A mRNA expression was determined by qRT-PCR (mean ± standard deviation of three independent experiments). **C**. Photograph of Aurora-A/phospho-T288 and Aurora-A protein expression by immunoblot analysis, representative for 3 independent experiments. β-Actin served as loading control.

For Aurora-A (Table 1, Figure 2), fluorescence *in situ *hybridization (FISH) revealed chromosome 20 polysomy with concomitantly elevated Aurora-A gene copy numbers in OE21, OE33 and OE19 cells and an Aurora-A gene amplification with up to nine Aurora-A gene copies in Kyse-410 cells. In view of their Aurora-A gene amplification, Kyse-410 cells also showed highest Aurora-A mRNA (qRT-PCR) and high protein (immunoblot) expression. In contrast, OE21, OE33 and OE19 cells exhibited lower Aurora-A mRNA expression, despite chromosome 20 polysomy. Still, high Aurora-A protein expression was seen in OE33, but not OE21 and OE19 cells. Active (phosporylated T288) Aurora-A was hardly detectable in immunoblot analysis, but weak Aurora-A/phosphoT288 levels were seen in OE21, Kyse-410 and OE33 cells. Control EPC-hTERT cells had normal diploid Aurora-A gene copy numbers, lowest Aurora-A mRNA expression, but detectable strong Aurora-A and weak Aurora-A/phosphoT288 protein levels.

**Table 1:** Aurora-A and -B gene copy numbers in normal esophageal epithelial and esophageal cancer cell lines.

		ESCC		BAC	
		
	EPC-hTERT	OE21	Kyse-410	OE33	OE19
**Aurora-A**					

Aurora-A gene copies	2.0 ± 0.2	4.2 ± 0.5	9.1 ± 1.2	7.5 ± 0.9	3.9 ± 0.3

Centromere 20 signals	1.9 ± 0.3	4.1 ± 0.7	4.6 ± 0.9	7.2 ± 0.9	2.9 ± 0.4

Aurora-A/CEP20 ratio	1.1 ± 0.3	1.1 ± 0.3	2.0 ± 0.4	1.1 ± 0.1	1.4 ± 0.2

**Aurora-B**					

Aurora-B gene copies	1.9 ± 0.4	3.0 ± 0.1	3.7 ± 0.8	2.9 ± 0.7	2.0 ± 0.2

Centromere 17 signals	2.0 ± 0.2	3.0 ± 0.1	3.8 ± 0.7	4.9 ± 0.9	3.9 ± 0.4

Aurora-B/CEP17 ratio	1.0 ± 0.2	1.0 ± 0.0	1.0 ± 0.1	0.6 ± 0.2	0.5 ± 0.1

The table summarizes results of FISH analyses of control esophageal epithelial cells (EPC-hTERT) and all four esophageal cancer cell lines, with mean and ± standard deviation of gene specific (Aurora-A, Aurora-B) and centromere (CEP, chromosome enumeration probe) specific FISH signals as well as the amplification status (gene to centromere ratio: Aurora-A to CEP20; Aurora-B to CEP17).

**Figure 3:**
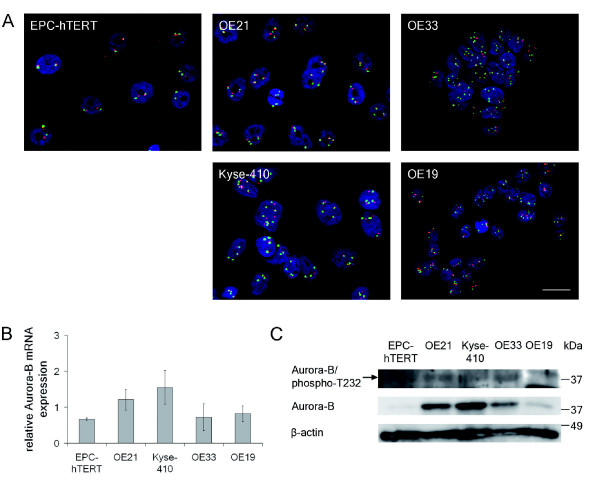
**Aurora-B gene copy numbers, mRNA and protein expression in normal esophageal epithelial cells and esophageal cancer cells**. **A**. FISH analysis of *AURKB *(red signals) and Chromosome 17 (CEP17; green signals). Bar = 20 μm, all panels are in the same magnification. **B**. Relative Aurora-B mRNA expression was determined by qRT-PCR (mean ± standard deviation of three independent experiments). **C**. Photograph of Aurora-B/phospho-T232 and Aurora-B protein expression by immunoblot analysis, representative for 3 independent experiments. β-Actin served as laoding control.

For Aurora-B (Table 1, Figure 3), chromosome 17 polysomy and concomitantly elevated Aurora-B gene copy numbers were observed by FISH in the ESCC cell lines OE21 and Kyse-410. Interestingly, in the BAC cell lines OE33 and OE19 elevated chromosome 17 specific signals with lower Aurora-B gene specific signals, resulting in Aurora-B to chromosome 17 ratios below 1, were observed. Accordingly, both ESCC (OE21, Kyse-410) cell lines had slightly higher Aurora-B mRNA and protein expression than the BAC cell lines (OE33, OE19). Active (phosphorylated T232) Aurora-B was apparent in OE21, Kyse-410 and OE33 cells. Control EPC-hTERT cells had normal diploid Aurora-B gene copy numbers, similar Aurora-B mRNA as BAC cell lines, but undetectable Aurora-B protein expression or activity.

The low Aurora-B gene copy numbers and protein expression in the two BAC cell lines were not due to a general phenomenon of entire chromosome 17 alterations, since (other than *AURKB *at 17p13) *HER2 *gene copy numbers (17q21) were highly amplified in these two cell lines (Supplementary figure 1).

Thus, Aurora-A and -B gene copy numbers are linked to mRNA expression patterns, but this is not directly translated into altered protein or activity levels. Whilst high Aurora-A and Aurora-B protein levels largely reflect DNA copy numbers as well as cell cycle distribution in some cell lines (OE21, Kyse-410), decoupling of Aurora-A and/or -B gene copy numbers with expression and cell cycle distribution occurs in other cell lines (particularly OE33).

### High Aurora-A expression alone is not associated with occurrence of multipolar mitoses in esophageal cancer cells

**Figure 4:**
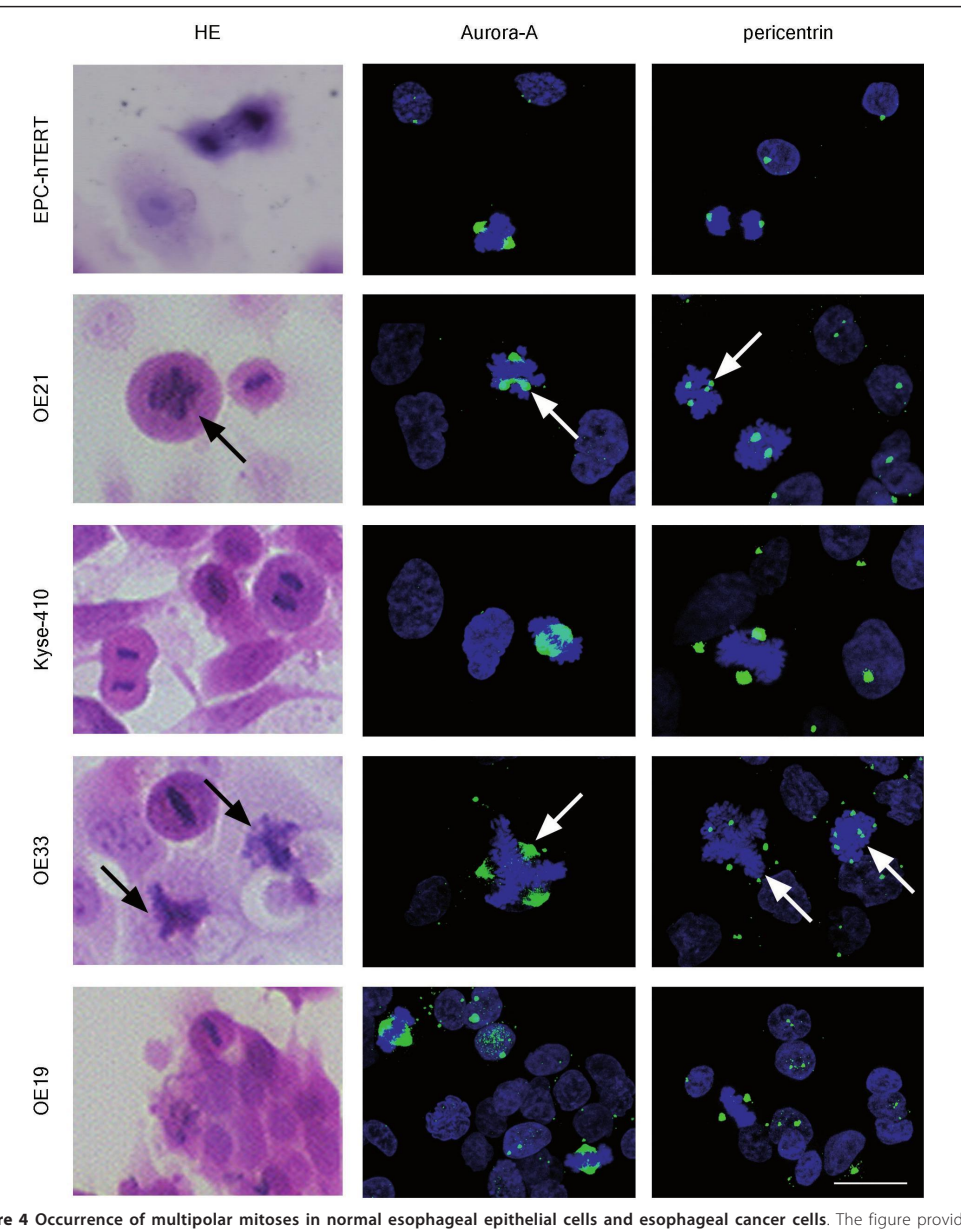
**Occurrence of multipolar mitoses in normal esophageal epithelial cells and esophageal cancer cells**. The figure provides representative pictures of HE stainings as well as Aurora-A and pericentrin indirect immunofluorescence analyses of EPC-hTERT, OE21, Kyse-410, OE33 and OE19 cell lines. Aurora-A staining (green) and pericentrin staining (green), each with counterstaining of DNA by DAPI (blue). Bar = 20 μm, all panels are in the same magnification. Note that the mitotic index and occurrence of multipolar mitoses were quantified according to Aurora-A indirect immunofluorescence of three independent experiments, with multipolar mitoses (arrows) most frequently observed in OE33 and OE21 cells (see Table 2 for quantification).

Aurora-A gene amplification and protein overexpression have been linked to the occurrence of supernumerary centrosomes, formation of multipolar mitoses and aneuploidy [27,30]. We therefore next examined the occurrence of Aurora-A positive multipolar mitoses in the EPC-hTERT as well as the four esophageal cancer cell lines. For this, three independent experiments were performed by quantitative, Aurora-A specific, indirect immunofluorescence [30] with asynchronized cells, to detect occurrence of mitoses and multipolar mitoses in view of their own cell cycle dynamics (Table 2, Figure 4). Parallel hematoxylin and eosin (HE) staining confirmed the data on mitotic cells morphologically and pericentrin-specific indirect immunofluorescence [14,30] confirmed the presence of Aurora-A associated supernumerary centrosomes.

**Table 2:** Mitotic index and occurrence of multipolar mitoses in normal esophageal epithelial cells and esophageal cancer cells.

A				ESCC						BAC					
	EPC-hTERT			OE21			Kyse-410			OE33			OE19		
	
Experiment	cells	mitoses	%	cells	mitoses	%	cells	mitoses	%	cells	mitoses	%	cells	mitoses	%
1	107	1	0.9	100	6	6.0	113	3	2.6	112	6	5.4	102	1	1.0

2	107	1	0.9	105	4	3.8	101	2	2.0	100	1	1.0	112	5	4.5

3	100	1	1.0	102	4	3.9	109	2	1.8	108	4	3.7	124	5	4.0

**mitotic index (Ø)**	**1.0 ± 0.0%**			**4.6 ± 1.2%**			**2.2 ± 0.4%**			**3.4 ± 2.2%**			**3.2 ± 1.9%**		

**B**				**ESCC**						**BAC**					
	**EPC-hTERT**			**OE21**			**Kyse-410**			**OE33**			**OE19**		
	
**Experiment**	**mitoses**	**multipolar mitoses**	**%**	**mitoses**	**multipolar mitoses**	**%**	**mitoses**	**multipolar mitoses**	**%**	**mitoses**	**multipolar mitoses**	**%**	**mitoses**	**multipolar mitoses**	**%**

1	81	0	0.0	100	13	13.0	101	8	7.9	104	19	18.3	101	2	2.0

2	100	1	1.0	100	7	7.0	100	7	7.0	100	10	10.0	101	1	1.0

3	84	0	0.0	100	3	3.0	100	4	4.0	100	13	13.0	100	0	0.0

**multipolar mitoses (Ø)**	**0.3 ± 0.6%**			**7.7 ± 5.0%**			**6.3 ± 2.0%**			**13.8 ± 4.2%**			**1.0 ± 1.0%**		

**(A) **Summary of the evaluation of the mitotic index. In three independent experiments, about 100 cells were counted and the number of mitoses therein was determined after Aurora-A immunofluorescence staining. The table also provides mean and ± standard deviation of the percentage of mitoses. **(B) **Summary of the evaluation of the index of multipolar mitoses. In three independent experiments, about 100 mitoses were counted and the number of multipolar mitoses therein was determined after Aurora-A immunofluorescence staining. The table also provides mean and ± standard deviation of the percentage of multipolar mitoses.

To specify the previous flow cytometric analyses (Figure 1), which only provided data on the total number of G2/M-phase cells, the mitotic index (i.e. only mitotic cells per total cells in %) was evaluated in indirect immunofluorescence analysis of Aurora-A and nuclear (DAPI) staining. For each cell line at least 100 cells were counted in three independent experiments (total at least 300 cells for each cell line; Table 2A, Figure 4). This revealed the highest mitotic index in OE21 (4.6 ± 1.2%), followed by OE33 (3.4 ± 2.25%), OE19 (3.2 ± 1.9%), Kyse-410 (2.2 ± 0.4%) and EPC-hTERT (1.0 ± 0.0%) cells.

Similarly, the occurrence of multipolar mitoses (i.e. multipolar mitoses per total mitoses in %) was assessed by quantifying indirect immunofluorescence analysis of Aurora-A and nuclear (DAPI) stainings. For this, in each cell line at least 80 mitoses were counted in three independent experiments (total at least 265 mitoses for each cell line; Table 2B, Figure 4). Aurora-A positive multipolar mitoses were most frequent in OE33 (13.8 ± 4.2%) followed by OE21 (7.7 ± 5.0%) and Kyse-410 (6.3 ± 2.0%) cells. OE19 cells (1.0 ± 1.0%) as well as EPC-hTERT cells (0.3 ± 0.6%), if any, only had single Aurora-A positive multipolar mitoses. Presence of supernumerary centrosomes in these multipolar mitoses was confirmed by pericentrin staining (Figure 4, right panels).

These data suggest that similarly high Aurora-A expression (as seen e.g. for Kyse-410 and OE33 cells) alone is insufficient to induce prominent multipolar mitoses in aneuploid esophageal cancer cells.

### Distinct p53 mutations contribute to multipolar mitoses in esophageal cancer cells

**Figure 5:**
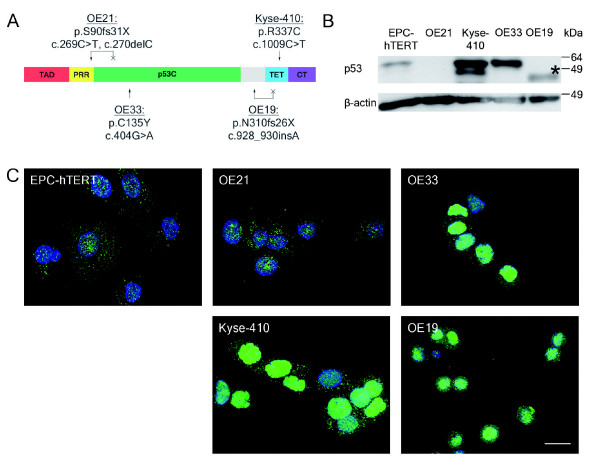
**p53 in normal esophageal epithelial cells and esophageal cancer cells**. **A**. The p53 protein and its domain structure (modified from [39]). The normal esophageal epithelium cell line EPC-hTERT carries no p53 mutation whereas all esophageal cancer cell lines exhibit mutations of p53, but each in a different functional domain. Mutations are indicated by arrows (↓) and possible protein truncations by crosses (×). **B**. Photograph of p53 protein expression by immunoblot analysis, representative for 3 independent experiments. Due to mutations, the p53 protein of OE21 and OE19 is truncated, resulting in protein masses of about 14 kDa and 40 kDa, respectively. For OE19 this shift in protein mass was seen at the immunoblot (asterisks), the 14 kDa protein of OE21 cells was below the size of detectable proteins in these 10% SDS gels. β-Actin served as laoding control. **C**. p53 indirect immunofluorescence (green) and DNA counterstaining by DAPI (blue). Mutated p53 accumulates in the nucleus of Kyse-410, OE33 and OE19 cells. The truncated p53 protein of OE21 cells lacks almost all protein domains and is only weakly expressed. Refer to text for discussion of p53 mutations.

In view of the role of p53 in post-mitotic cell cycle control, centrosome duplication and Aurora-A interaction [41-45,53] as well as its frequent mutation in esophageal carcinogenesis [4,10,11], we next determined p53 mutation status [54,55], p53 protein expression and intracellular localization [39] in the control EPC-hTERT cell line and in the four esophageal cancer cell lines (Figure 5).

The control EPC-hTERT cells exhibited a wild type p53 sequence and showed weak p53 protein expression in immunoblot (Figure 5B) and indirect immunofluorescence analysis (Figure 5C). This wild type p53 protein was located in the cytoplasm of EPC-hTERT cells.

In contrast, all ESCC and BAC cell lines displayed p53 mutations (Figure 5A): OE21 cells exhibited p53 mutations in exon 4 (c.269C>T, c.270delC, p.S90fs31X), which introduce a stop codon at the N-terminus of the p53 core domain. The p53 protein of OE21 cells lacks almost the entire DNA binding domain, the tetramerization domain and the extreme C-terminus (protein mass of truncated protein is about 14 kDa). This protein, if at all being expressed, is most likely non-functional since almost all domains are missing, including the Aurora-A interaction sites Serine 215 and 315. Indeed, immunoblot analysis did not detect this largely truncated p53 protein (Figure 5B) and immunofluorescence showed only weak and rather diffusely localized p53 staining in OE21 cells (Figure 5C).

Kyse-410 cells displayed a point mutation in exon 10 (c.1009C>T, p.R337C) of the tetramerization domain (Figure 5A). This mutation was neither found in the original human cancer, of which this cell line was derived from, nor in the original Kyse-410 cell line [56]. However, it was later reported in Kyse-410 cells [57]. There are conflicting reports about whether this mutated p53 protein forms tetramers, binds DNA, induces apoptosis and transactivates target genes or not. It seems that p53 with this mutation is partially functional depending on the experimental conditions [58-60]. In our case, this mutated p53 protein was clearly detectable in immunoblot analysis (Figure 5B) and displayed a strong nuclear staining in most, but not all Kyse-410 cells by indirect immunofluorescence (Figure 5C).

OE33 cells had a point mutation in exon 5 (c.404G>A, p.C135Y), which is consistent with previous reports [57] (Figure 5A). This mutation abolishes the p53 transactivation activity as well as growth suppressive activity of the mutated protein and has a dominant negative effect on wild type p53 [61,62]. Accordingly, this mutated p53 protein was still expressed and accumulated in OE33 cell nuclei, although in some cells to a weaker extent (Figure 5B, C).

OE19 cells exhibited a mutation in exon 9 (c.928_930insA, p.N310fs26X), which is in accordance with mutation databases [57]. This mutation is within the flexible linker, which connects the p53 core domain with the tetramerization domain, causes a stop codon within the tetramerization domain (protein mass of truncated protein is about 40 kDa) and most likely inactivates p53 oligomerization (Figure 5A). However, the latter is insufficient to fully abolish p53 tumor suppressive function and p53 monomer mutants with retention of transcriptional activity have been described [58]. In OE19 cells, this potentially still functional mutated p53 protein was strongly expressed as truncated protein at 40 kDa in immunoblot analysis (Figure 5B, asterisks) and clearly accumulated in OE19 cell nuclei (Figure 5C).

Thus, loss of function p53 mutations may result in escape of post-mitotic G1 cell cycle control and possibly also centrosomal dysfunction [41-45] in some (OE21, OE33), but not all (Kyse-410, OE19) esophageal cancer cells.

## Discussion

This study addressed Aurora kinases A and B, p53 mutations and occurrence of multipolar mitoses in aneuploid esophageal squamous cell carcinoma (ESCC) and Barrett's adenocarcinoma (BAC) cell lines (for data summary refer to Table 3).

**Table 3:** Summary of investigated parameters in normal esophageal epithelial cells and esophageal cancer cell lines.

		ESCC		BAC	
	EPC-hTERT	OE21	Kyse-410	OE33	OE19
**Aurora-A**					

Aurora A gene copies	2.0 ± 0.2	4.2 ± 0.5	9.1 ± 1.2	7.5 ± 0.9	3.9 ± 0.3

Centromere 20 signals	1.9 ± 0.3	4.1 ± 0.7	4.6 ± 0.9	7.2 ± 0.9	2.9 ± 0.4

Aurora-A mRNA	0.4 ± 0.1	0.8 ± 0.1	2.7 ± 0.3	1.3 ± 0.3	1.6 ± 0.1

Aurora-A protein	++	+	+++	+++	+

Aurora-A/phospho T288	+	+	+	(+)	(+)

**Aurora-B**					

Aurora-B gene copies	1.9 ± 0.4	3.0 ± 0.1	3.6 ± 0.8	2.9 ± 0.7	2.0 ± 0.2

Centromere 17 signals	2.0 ± 0.2	3.0 ± 0.1	3.8 ± 0.7	4.9 ± 0.7	3.9 ± 0.4

Aurora A mRNA	0.7 ± 0.0	1.2 ± 0.3	1.5 ± 0.5	0.7 ± 0.4	0.8 ± 0.2

Aurora-B protein	(+)	+++	++++	++	+

Aurora-B/phospho T232	(+)	+	+	+	-

**p53**					

Mutations	no mutation	c.269C>T, c.270delC, p.S90fs31X	c.1009C>T, p.R337C	c.404G>A, p.C135Y	c.928_930insA, p.N310fs26X

p53 protein (kDa)	53	~14 (truncated)	53	53	~40 (truncated)

p53 functionality	+	-	+/-	+	+/-

**Mitoses**					

Mitotic Index (%)	1.0 ± 0.0%	4.6 ± 1.2%	2.2 ± 0.4%	3.4 ± 2.2%	3.2 ± 1.9%

Multipolar mitoses (%)	0.3 ± 0.6%	7.7 ± 5.0%	6.3 ± 2.0%	13.8 ± 4.2%	1.0 ± 1.0%

Quantitative data of each three independent experiments are summarized for FISH and q-RT-PCR (relative mRNA expression) analyses as well as for mitoses and multipolar mitoses (mean and ± standard deviation). Immunoblot analyses are depicted with minus (-) to increasingly positive (+ to ++++) protein expression. Identified p53 mutations and associated effects are listed.

Amplification of 20q13 and/or Aurora-A has been reported to occur frequently in human esophageal carcinomas by extract-based methods, such as (array-based) comparative genomic hybridization [24,34,36,37]. The present study confirms the importance of this chromosomal region in ESCC and BAC, but our precise single cell FISH analyses of each two ESCC and BAC cell lines suggests that high level Aurora-A gene amplification is a rather rare event in esophageal cancer cells. A clear-cut Aurora-A gene amplification was only seen in Kyse-410 cells, as described before [24], whilst all other investigated cell lines had increased Aurora-A gene copy numbers due to chromosome 20 polysomy. Moreover, elevated Aurora-A gene copy numbers may not necessarily result in elevated Aurora-A mRNA and/or protein expression, as exemplified by our results of OE21 and OE19 cells. Also, Aurora-A gene copy numbers are far from a direct link to activated Aurora-A (phosphorylated at T288) protein levels. Whilst cell cycle dynamics of esophageal cancer cells clearly impact detection levels of Aurora-A expression, additional regulation of Aurora-A expression by transcriptional, for example via epidermal growth factor receptor [63], and post-translational, for example via the ubiquitin-proteasome pathway [64], mechanisms may further act in individual esophageal cancer cells. Indeed, the clinical relevance of Aurora-A in esophageal cancers has mainly been determined at the expression level [20,22,23].

In contrast to Aurora-A, there was a more close association between Aurora-B gene copy numbers and Aurora-B mRNA and protein expression in the ESCC and BAC cell lines. Both ESCC cell lines (OE21, Kyse-410) had elevated Aurora-B gene copy numbers due to chromosome 17 polysomy and concomitant high Aurora-B expression, but not activation (phosphorylation at T232). Instead, both BAC cell lines (OE33, OE19) displayed lower Aurora-B gene specific signals than chromosome 17 specific signals with concomitantly low Aurora-B mRNA as well as protein expression and activity. In fact, also our previous studies showed broad chromosomal deletions on 17p close to the Aurora-B locus in up to 40% of tissue specimens of BACs [36,37], whilst other investigators reported controversial results for chromosome 17p alterations in tissue specimens of ESCC [34,35]. In order to rule out that this is due to a major chromosome 17 alteration, we performed FISH and immunoblot analysis for HER2 (17q21), clearly demonstrating that HER2 is highly amplified in these two BAC cell lines. This suggests that the detected genomic alteration is specific to 17p, respective potentially the Aurora-B gene. The apparently "reduced" Aurora-B gene copy numbers in BAC cells may be due to a partial deletion, loss of the short arm of chromosome 17 or even duplication of centromere 17 alone. It will be of interest for future studies to investigate potentially deregulated chromosome integrity, for example by telomere alterations or breakage-fusion-bridge cycles, during mitosis of BAC cells.

Irrespective of this, the present results allow further insights into the direct association of high Aurora-A expression with supernumerary centrosomes and the associated occurrence of multipolar mitoses and aneuploidy described in other model systems [27,30] now also for aneuploid esophageal cancer cells. For example, ectopic overexpression of functional Aurora-A in diploid colorectal cancer cell line [31] or ectopic expression of kinase deficient Aurora-A isoforms, which is unable to phosphorylate its substrate Lats2, in immortalized fibroblasts [32] both resulted in either supernumerary centrosomes, chromosome segregation defects and/or genomic instability.

In fact, we found that high Aurora-A expression in aneuploid ESCC or BAC cells does not determine the occurrence of multipolar mitoses alone: 1) although exhibiting similarly high Aurora-A expression, only OE33 cells, but not Kyse-410 cells were characterized by a high frequency of multipolar mitoses and 2) of the two cell lines with markedly lower Aurora-A expression (OE21, OE19), OE21 cells did in fact show a noticeable frequency of multipolar mitoses, being higher than in strongly Aurora-A expressing Kyse-410 cells.

Thus, other factors are required to sustain cycling of multipolar mitotic esophageal cancer cells in order to allow development and maintenance of individual aneuploid ESCC and BAC cell clones. Since up to 80% of ESCC and 90% of BAC display mutations of p53 [4,10,11], this tumor suppressor protein is a very likely contributing factor, particularly in view of its role in G1 cell cycle and DNA damage control, its centrosomal function [39-43] as well as its inactivation and/or degradation upon interaction with Aurora-A [44,45]. Thus, cells with still intact or only partial dysfunctional p53 protein may still have p53 dependent G1 cell cycle control, a scenario that was of interest for the present data, particularly for the ESCC Kyse-410 cells.

In the present study, p53 mutations were found in the four representative esophageal cancer cell lines, but in different domains and therefore with different consequences for protein expression and/or function. None of the p53 mutations detected corresponded to known p53 gain of function mutations [65]. Instead, OE21 cells had p53 mutations (exon 4; c.269C>T, c.270delC, p.S90fs31X), which caused weak expression of a presumably non-functional, largely truncated (about 14 kDa) p53 protein. Also OE33 cells had a p53 mutation (exon 5; c.404G>A, p.C135Y) resulting in a non-functional, nuclear accumulated p53 protein, lacking transactivation and growth suppressive activity [61,62]. In contrast, Kyse-410 cells had a cell culture acquired [56,57] p53 mutation (exon 10; c.1009C>T, p.R337C), resulting in expression and nuclear accumulation of an at least partially functional p53 protein [58-60]. Similarly, the p53 mutation confirmed in OE19 cells (exon 9; c.928_930insA, p.N310fs26X) [57], may cause expression of a truncated (about 40 kDa), but still partially functional p53 protein with lost oligomerization activity [58]. Thus, esophageal cancer cells with both high Aurora-A expression and p53 loss of function mutations have a high occurrence of multipolar mitoses (e.g. OE33: about 14% multipolar mitoses). In contrast, esophageal cancer cells with Aurora-A gene amplification and high Aurora-A expression, but an at least partially functional p53 protein have fewer multipolar mitoses (e.g. Kyse-410: about 8% multipolar mitoses).

In contrast to the esophageal cancer cells, the normal esophageal epithelial cell line EPC-hTERT [50,51] was diploid, had wild type p53 and did show normal Aurora-A and Aurora-B gene copy numbers as well as bipolar mitoses. Still, slightly elevated Aurora-A and p53 protein levels were observed in this cell line. Although no effect of hTERT was seen on p16 and p53 protein levels in the initial description of this cell line [51], others have reported an effect of hTERT-induced downregulation of p16, p21 and up-regulation of Aurora-A in normal esophageal epithelial cells [66], which may explain the detectable Aurora-A protein expression observed in our experiments of EPC-hTERT cells.

## Conclusions

In summary, high Aurora-A expression together with p53 mutations may contribute to aneuploid esophageal cancer cells via supernumerary centrosomes and associated occurrence of multipolar mitoses. This is heterogeneous in single ESCC or BAC cell lines, thereby reflecting the heterogeneity also observed in individual patients with ESCC or BAC [20-25]. The study therefore represents a basis for further translational assessment of Aurora kinases and associated cell cycle control in aneuploid ESCC and BAC cells, particularly also in view of discussions of Aurora kinases as therapeutic targets [17,67,68]. Further assessment of Aurora kinases and p53 interactions in cell lines or tissue specimens derived from precursor lesions of dysplasia (for ESCC) or intestinal metaplasia (for BAC) are necessary to disclose a causative role of Aurora kinases and p53 in the development of aneuploid, invasive esophageal cancers.

## Methods

### Cell culture

The study included as control a normal esophageal epithelial cell line (EPC-hTERT cells) [50,51] as well as four esophageal cancer cell lines (OE21, Kyse-410, OE33, OE19). The esophageal cancer cell lines were originally derived from patients with esophageal squamous cell carcinomas (ESCC: OE21, Kyse-410), Barrett's adenocarcinoma (BAC: OE33) or an esophageal junctional adenocarcinoma (OE19) [47,48]. Indeed, the specificity of the adenocarcinoma cell lines was recently approved [49]. Due to clear adenocarcinoma differentiation and growth patterns, the two cell lines OE33, OE19 are collectively referred to as "BAC" in the present *in vitro *study, which does not address the carcinogenesis of esophageal carcinomas in view of the intestinal metaplasia-dysplasia-carcinoma sequence.

EPC-hTERT cells were cultivated in Keratinocyte-SFM medium (Invitrogen, Karlsruhe, Germany) supplemented with 40 μg/ml bovine pituitary extract (Invitrogen, Karlsruhe, Germany), 1.0 ng/ml EGF (Invitrogen, Karlsruhe, Germany), 100 units/ml penicillin and 100 μg/ml streptomycin (Invitrogen, Karlsruhe, Germany) at 37°C in a 5% CO2 atmosphere.

The esophageal cancer cell lines OE21 and Kyse-410 and the BAC cell lines OE33 and OE19 (European Collection of Cell Cultures, Salisbury, UK) were cultivated in RPMI 1640 medium (PAA Laboratories, Pasching, Austria), supplemented with 10% (v/v) Fetal Bovine Serum (PAA Laboratories, Pasching, Austria) and 2 mM GIBCO™ L-Glutamin (Invitrogen, Karlsruhe, Germany) at 37°C in a 5% CO2 atmosphere.

### Hematoxylin and Eosin staining (HE staining)

Cells grown on coverslips were fixed with 4% paraformaldehyde (PFA), rinsed with Phosphate buffered saline (PBS: 137 mM NaCl, 10 mM Na_2_HPO_4_, 1.8 mM KH_2_PO_4_; pH 6.8-7) and stained with Hematoxylin (Carl Roth, Karlsruhe, Germany). After removing the hematoxylin solution mains water was added twice. Cells were stained with Eosin Y solution (Carl Roth, Karlsruhe, Germany) and distilled water was added. The coverslips were then immersed in an ascending ethanol series and in xylol.

### Cell cycle phase distribution analysis by flow cytometry

For cell cycle distribution analyses by flow cytometry cells were grown to 50%-60% confluency. The cells in the medium and trypsinized cells were collected and fixed in ice-cold 70% (v/v) ethanol. After washing with PBS cells were stained with propidium iodide (20 μg/ml propidium iodide (Sigma-Aldrich, Steinheim, Germany), 0.1% (v/v) Tritron X-100, 0.2 mg/ml Ribonuclease A (Sigma-Aldrich, Steinheim, Germany) in PBS). Stained cells were analyzed using the LSRII system and DB FACS Diva software (Becton Dickinson, Heidelberg, Germany).

### Fluorescence *in situ *hybridization (FISH)

Cells were grown on Poly-L-Lysine coated Lab-Tek^® ^1 Well Glass Slides (Thermo Fisher Scientific, Langenselbold, Germany). Cells were washed with PBS, fixed in 3:1 methanol/glacial acetic acid and dehydrated in an ethanol series. *AURKA *(20q13) & 20q11 DNA probe (Kreatech Diagnostics, Amsterdam, Netherlands) or *AURKB *(17p13)/Alphasatellite 17 specific DNA probe (MP Biomedicals, Illkirch, France) was applied. Co-denaturation was performed for 5 min at 75°C for *AURKA *or 80°C for *AURKB *probes and hybridization for 16-18 h at 37°C in a humidified chamber. After washing in 0.4× SSC/0.3% (v/v) NP-40 pH 7 for 2 min at 73°C and in 2× SSC/0.1% (v/v) NP-40 pH 7-7.5 for 1 min at room temperature (RT), cell nuclei were counterstained with DAPI (Vector Laboratories, Burlingame, USA). Examination was done at a fluorescence microscope (Axioplan2 imaging microscope equipped with a Plan-Apochromat 63×/1.4 oil objective, Carl Zeiss MicroImaging, Göttingen, Germany) with slider module. Image stacks at 0.9 μm intervals were taken of at least three representative fields per cell line. Image stacks were converted into 3D view by AxioVision software (Carl Zeiss MicroImaging, Göttingen, Germany).

For each cell line, the gene (*AURKA *or *AURKB*) and chromosome specific signals (centromer enumeration probes/CEP; CEP20 or CEP17) were counted per individual cell nucleus (range of cell nuclei counted: 49-88; a mean of 71.3 ± 13.1 cell nuclei per cell line). The mean and standard deviation of the gene (*AURKA *or *AURKB*) and chromosome specific signals (CEP20 or CEP17) of counted cell nuclei were calculated for each cell line. The FISH ratio (*AURKA *to CEP20 and *AURKB *to CEP17) was calculated for each analyzed cell nucleus and thereof the mean and standard deviation was calculated for each cell line. True gene-specific amplification was considered at a FISH ratio of >2. The FISH procedure and quantification has previously been published by us for evaluation of Aurora-A and other gene copy numbers in tissue specimens [18].

### Indirect immunofluorescence and evaluation of mitoses

Cells were grown on coverslips, fixed in 2% PFA, washed in PBS and permeabilized in 0.5% (v/v) Tritron X-100 in PBS. After PBS washing, cells were incubated with blocking buffer (PBS containing 5.0% (v/v) normal goat serum and 0.3% (v/v) Tritron X-100). Diluted primary antibodies (mouse anti-IAK1/Aurora-A Kinase, 1:100, clone 4, BD Biosciences, Heidelberg, Germany; mouse anti-pericentrin, 1:1000, clone mAbcam 28144, Abcam, Cambridge, UK; mouse anti-p53, 1:50, clone DO-7, DakoCytomation, Hamburg, Germany) were incubated over night at 4°C, cells were rinsed with PBS and 1:200 diluted fluorescently labelled secondary antibodies (goat-anti-mouse IgG-Alexa488, Invitrogen, Karlsruhe, Germany), were incubated for 1 h at RT. After washing with PBS and distilled water, cell nuclei were counterstained with DAPI (Vector Laboratories, Burlingame, USA). Note that the p53 antibody used was raised against the N-terminal domain (amino acids 1-45), recognizing also mutated and expressed (also truncated) p53 proteins.

Normal bipolar mitoses were defined as mitotic cells with 2 Aurora-A positive centrosomes/spindle poles. Multipolar mitoses were defined as mitotic cells with >2 Aurora-A positive centrosomes/spindle poles. In three independent experiments, cells were screened using a x40 objective and a minimum of 100 cells were counted for the mitotic index (mitoses per cells counted in %; range of total cells counted: 100-124) and up to 100 mitoses per cell line were evaluated for the occurrence of multipolar mitoses (multipolar mitoses per mitoses counted; range of total mitoses counted: 81-104) (Table 2).

### Immunoblotting

Preparation of total protein and determination of protein concentration was performed using the Qproteome™ Mammalian Protein Prep Kit (Qiagen, Hilden, Germany) and the DC Protein Assay (Bio-Rad, München, Germany) according to the manufacturer's protocols. 10 μg of total protein extracts per lane were loaded onto 10% polyacrylamide gels. Proteins were transferred onto Protran^® ^Nitrocellulose Transfer Membrane (Whatman, Dassel, Germany) by Semi-Dry Blot. After blocking the membrane in 5% (m/v) nonfat dried milk powder in Tris buffered saline with Tween (TBST: 10 mM Tris-Base, 150 mM NaCl, 0.1% (m/v) Tween; pH 7.2-7.4), the primary antibodies diluted in 5% (m/v) nonfat dried milk powder in TBST (mouse anti-IAK1/Aurora-A Kinase, 1:250, clone 4, BD Biosciences, Heidelberg, Germany; rabbit anti-Aurora-B, 1:5000, clone EP1009Y. Epitomics, Burlingame CA, USA; mouse anti-p53, 1:1000, clone DO-7, DakoCytomation, Hamburg, Germany; mouse anti-β-Actin, 1:1000, clone AC-15, Sigma-Aldrich, Steinheim, Germany) or 3% BSA in TBST (rabbit anti Aurora-A/phospho-T288, 1:1000, clone C39D8, Cell signalling, Danvers MA, USA) or 5% BSA in TBST (rabbit anti-Aurora-B/phospho-T232, 1:500, Abcam, Cambridge, UK) were incubated. After HRP conjugated secondary antibody (1:25000, Dianova, Hamburg, Germany) incubation, the membrane was incubated with ECL reagents (GE Healthcare, Freiburg i. Br., Germany) and exposed to autoradiography films. Note that the p53 antibody used was raised against the N-terminal domain (amino acids 1-45), recognizing also mutated and expressed (also truncated) p53 proteins.

### p53 mutation analysis

Genomic DNA was isolated using the QIAamp^® ^DNA Micro Kit (Qiagen, Hilden, Germany) according to the manufacturer's instruction. Amplification of p53 exons 2-11 was performed using primers and protocols slightly modified from previous studies [54,55]. PCR was carried out in a 25 μl reaction mixture containing 1× PCR Buffer, 1.5-2.5 mM MgCl2, 12 ng/μl gDNA, 0.4 mM dNTP Mix, 0.4 μM forward and reverse primers and 1.25 U Taq DNA polymerase. The PCR was performed with the following conditions: 94°C for 4 min, 40 cycles consisting of 94°C for 30 sec, 53-65°C for 30 sec and 72°C for 30 sec, followed by 72°C for 7 min. PCR products were purified using the QIAquick^® ^PCR Purification Kit (Qiagen, Hilden, Germany) according to the manufacturer's protocol. Sequencing was performed using BigDye^® ^Terminator v1.1 Cycle Sequencing Kit (Applied Biosystems, Darmstadt, Germany) according to the manufacturer's instruction. The reactions were performed in 20 μl reaction mixture consisting of 3-5 ng PCR product, 0.16 μM forward or reverse primers, 20% (v/v) BigDye^® ^Ready Reaction Mix and 1× Big Dye^® ^Sequencing Buffer. A positive control with a 20 μl reaction mixture containing 5% (v/v) pGEM®-3Zf(+) double-stranded DNA control Template, 5% (v/v) -21 M13 Control Primer (forward), 20% (v/v) BigDye^® ^Ready Reaction Mix and 1× Big Dye^® ^Sequencing Buffer was included. The PCR was performed with the following conditions: 96°C for 1 min, 24 cycles consisting of 96°C for 10 sec, 50°C for 5 sec and 60°C for 4 min. DNA was precipitated with ethanol containing 5 mM EDTA and 120 mM sodium acetate, dissolved in formamide and denatured for 5 min at 95°C. Capillary electrophoresis was performed using the ABI PRISM™ 310 Genetic Analyzer (Applied Biosystems, Darmstadt, Germany). The Sequencing Analysis Software V 5.2 (Applied Biosystems, Darmstadt, Germany) was used to analyze the collected electropherogram traces and sequencing information. The p53 sequence of the GenBank database with accession number NC_000017.9|NC_000017:c7531642-7512445 was used as reference.

### RNA isolation and cDNA synthesis

Total RNA isolation was performed using the RNeasy^® ^Mini Kit (Qiagen, Hilden, Germany) according to the manufacturer's instruction. For cDNA synthesis, a 9 μl reaction mixture containing 200 ng total RNA, 1 μl yeast RNA (10 ng/μl) and 2 μl Hexanucleotide Mix (10×, Roche Diagnostics, Mannheim, Germany) was incubated for 2 min at 70°C and 10 min at RT. A second 11 μl reaction mixture containing 4 μl First Strand Buffer (5×, Invitrogen, Karlsruhe, Germany), 2 μl DTT (0.1 M), 1 μl dNTP Mix (10 mM) and 1 μl M-MLV RT (200U/μl, Invitrogen, Karlsruhe, Germany), was added and incubated for 1 h at 37°C. The M-MLV RT was inactivated for 5 min at 95°C. For reverse transcription of Universal Human Reference RNA (uRNA) (Stratagene, Heidelberg, Germany) [69], the calibrator of qRT-PCR, 300 ng RNA was employed in an appropriate volume.

### Quantitative reverse transcription PCR (qRT-PCR)

qRT-PCR was performed using established protocols for Aurora-A and TBP [18]. Following Aurora-B primers and probes were used (sequence 5'-3'): Aurora-B-forward (900 nM): CAT GAG CCG CTC CAA TGT C, Aurora-B-reverse (50 nM): CCC AAT CTC AAA GTC ATC AAT TGT, Aurora-B-probe (150 nM): 6-FAM-ACA CCC GAC ATC TTA ACG CGG CA-TAMRA. The comparative Ct method was used to calculate Aurora-A/-B mRNA levels. The amount of the target gene is normalized to the endogenous housekeeping gene TATA box binding protein (TBP) and these relative fold differences are compared between the experimental and the uRNA calibrator sample [69,70].

## Abbreviations

BAC: Barrett's adenocarcinoma; CEP: centromere enumeration probe; ESCC: Esophageal squamous cell carcinoma; FISH: Fluorescence *in situ *hybridization; HE: Hematoxylin and Eosin staining; INCENP: Inner centromere protein; MDM2: Murine double minute-2; M-MLV RT: Moloney Murine Leukemia Virus Reverse Transcriptase; qRT-PCR: Quantitative reverse transcription PCR; TBP: TATA box binding protein; uRNA: Universal Human Reference RNA

## Authors' contributions

CDF carried out the experimental work, analysed and interpreted data, prepared figures and tables, drafted and revised the manuscript.

CH assisted in qRT-PCR and p53 mutation analyses, and interpreted data.

CM participated in FISH analysis and interpreted data.

OGO interpreted data, approved manuscript.

MW supervised study, interpreted data and approved manuscript.

SL designed and supervised the study, analyzed and interpreted data, wrote, revised and approved the manuscript.

## Authors' information

None

## Supplementary Material

Additional file 1 **Supplementary Figure S1: *HER2 *gene copy numbers in esophageal cancer cells**. FISH analysis of *HER2 *(red signals) and Chromosome 17 (CEP17; green signals). All panels are in the same magnification. Note HER2 gene amplification in OE33 and OE19 cells and chromosome 17 polysomy in all cell lines. Click here for file
