# Supplementary figures and images for: Occurrence of multipolar mitoses and association with Aurora-A/-B kinases and p53 mutations in aneuploid esophageal carcinoma cells
Source: BMC Cell Biol. 2011 Apr 6;12:13. doi: 10.1186/1471-2121-12-13 (PMC3094318; doi:10.1186/1471-2121-12-13)

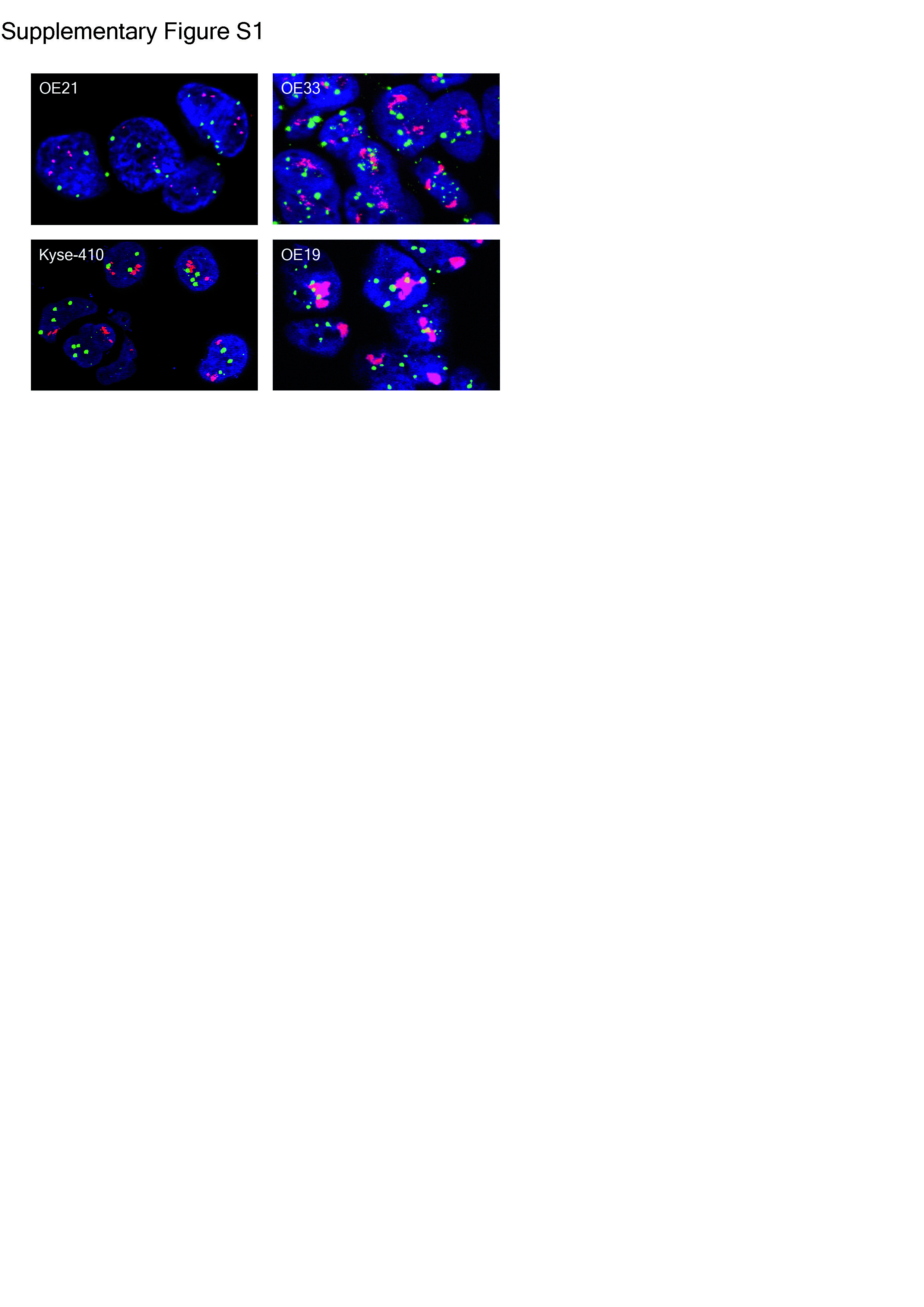

Supplement: Additional file 1 — Supplementary Figure S1: HER2 gene copy numbers in esophageal cancer cells. FISH analysis of HER2 (red signals) and Chromosome 17 (CEP17; green signals). All panels are in the same magnification. Note HER2 gene amplification in OE33 and OE19 cells and chromosome 17 polysomy in all cell lines. [file 1471-2121-12-13-S1.jpeg]
